# Contrasting reproductive traits of competing parasitoids facilitate coexistence on a shared host pest in a biological control perspective

**DOI:** 10.1002/ps.6965

**Published:** 2022-05-26

**Authors:** Antonino Cusumano, Ezio Peri, Tuğcan Alınç, Stefano Colazza

**Affiliations:** ^1^ Department of Agricultural, Food and Forest Sciences University of Palermo Viale delle Scienze Palermo Italy; ^2^ Interuniversity Center for Studies on Bioinspired Agro‐Environmental Technology (BAT Center) University of Napoli Federico II Portici Italy

**Keywords:** interspecific competition, extrinsic competition, intrinsic competition, egg parasitoids, stink bugs, *Trissolcus basalis*, *Ooencyrtus telenomicida*, *Nezara viridula*

## Abstract

**BACKGROUND:**

Interspecific competition in insect parasitoids is an important ecological phenomenon that has relevant implications for biological pest control. To date, interspecific intrinsic (=larval) competition has been intensively studied, while investigations on extrinsic (=adult) competition have often lagged behind. In this study we examined the role played by parasitoid reproductive traits and host clutch size on the outcome of extrinsic competition between *Trissolcus basalis* (Wollaston) and *Ooencyrtus telenomicida* (Vassiliev), two egg parasitoids of the pest *Nezara viridula* (L). Laboratory experiments were conducted by allowing both parasitoid species to exploit an egg mass made of 10, 20, 30, or 40 hosts through single or simultaneous releases. Furthermore, under field conditions, egg masses consisting of 10 or 40 hosts were exposed in a tomato crop in order to validate laboratory investigation.

**RESULTS:**

The results show that the egg mass size is an important predictor of extrinsic competition in our study system as a higher proportion of *T. basalis* emerged from large egg masses, while *O. telenomicida* dominated in small egg masses. Analysis of reproductive traits of parasitoid species indicates that *T. basalis* has superior abilities in host exploitation compared with *O. telenomicida*.

**CONCLUSIONS:**

We found that contrasting reproductive traits of two competing egg parasitoid species facilitate coexistence on a shared stink bug host. This work also highlights the importance to consider extrinsic competitive interactions between parasitoid species in a biological control perspective. © 2022 The Authors. *Pest Management Science* published by John Wiley & Sons Ltd on behalf of Society of Chemical Industry.

## Graphical abstract

This study examined the role played by parasitoid reproductive capacities and host clutch size on the outcome of extrinsic competition between *Trissolcus basalis* and *Ooencyrtus telenomicida*, two egg parasitoids of the pest *Nezara viridula* . Results showed that contrasting reproductive capacities of two competing egg parasitoid species facilitate coexistence on a shared stink bug host.

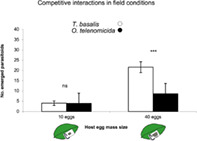

## 1 INTRODUCTION

When foraging for suitable hosts, insect parasitoids can engage in complex interactions with other parasitoid species, hyperparasitoids, predators, and entomopathogens.^1^, ^2^, ^3^ The interspecific interactions between insect parasitoids can be particularly severe as parasitic wasps may employ similar foraging strategies and exploitation modes leading to intense competition for the shared host resources.^4^, ^5^ Yet despite strong competition, several parasitoid species can co‐occur on a single host indicating that parasitoid species have established highly specific behavioral and physiological mechanisms to reduce the degree of niche overlap in order to minimize competition for hosts.^6^ There are many ways in which parasitoid niches may diverge among competing species: for example, parasitoids can have different host breadth, *i.e*. generalists *versus* specialists, or parasitoids with a similar degree of host specialization can search for hosts in different habitats or at different times.^7^, ^8^, ^9^

Interspecific competition between parasitoids can play a role in species coexistence and in sizing and shaping community structures.^10^ In particular, interspecific competition for host resources is an important ecological phenomenon which should be considered in biological control programs since it may affect the efficiency of the biological control agent, the establishment of a novel species in classical biocontrol programs and it may even lead to the replacement of one parasitoid species by another one.^11^, ^12^, ^13^ Interspecific competition in parasitoid species can be classified into two categories: (i) extrinsic competition i.e., interactions between adult females searching for or exploiting hosts, and (ii) intrinsic competition i.e., interactions between parasitoids larvae developing in the same host. The outcome of extrinsic competition can be mediated by differences in host‐seeking strategies, dispersal capacities, aggressive behaviour, reproductive potential and oviposition abilities of female parasitoids and phenological synchronization with the host,^14^, ^15^, ^16^, ^17^ while intrinsic competition is mediated by physical or physiological suppression between immature parasitoid larvae developing in the same host.^18^, ^19^, ^20^, ^21^, ^22^ To date, intrinsic interactions have been intensively studied and documented whereas investigations on extrinsic competition among parasitoid species have often lagged behind.^5^ One reason for this may lie in the fact that studying extrinsic competition among adult parasitoids is more complex than investigating intrinsic competition among parasitoid larvae. In fact, while it is relatively straightforward to identify the superior intrinsic competitor by observing the outcome of larval competition, it is far more challenging to investigate which species has superior extrinsic competitive abilities.^10^ Yet it is becoming increasingly evident that a clear understanding of both extrinsic and intrinsic competition is needed in order to explain species co‐existence and elucidate the role of interspecific competition in biological control programs.^5^

In some case studies, it has been demonstrated that the parasitoid species most negatively affected by intrinsic competition is the most effective biological control agent.^23^, ^24^, ^25^ The first evidence of this outcome was shown by Force.^23^ In his highly influential work, Force^23^ manipulated, in confined environments, populations of gall midge *Rhopalomyia californica* Felt by introducing different combinations of six natural parasitoid species. He was able to assess the impact of these combinations on the host population concluding that the parasitoid species with the poorest intrinsic competitive abilities, but highest reproductive abilities (*i.e. Tetrastichus* sp.) was capable of achieving the higher parasitism rate when acting alone.

Extrinsic and intrinsic interactions have been investigated in *Trissolcus basalis* (Wollaston) and *Ooencyrtus telenomicida* (Vassiliev), two parasitoids that compete for eggs of the Southern Green Stink Bug, *N. viridula* (L.).^26^ This stink bug species is a serious pest of several fresh vegetables including tomato where insect feeding on fruits cause discoloration upon ripening and development of corky area below the fruit surface.^27^

In the field, the parasitoids *T. basalis* and *O. telenomicida* can naturally co‐occur competing on the same host.^28^ It has been shown, under laboratory conditions, that the former is extrinsically superior when considering host finding abilities.^26^, ^29^ Comparative olfactometer and open arena bioassays have shown that *T. basalis* females are more efficient in host location as they use volatile synomones induced by egg deposition, volatile and contact kairomones;^30^, ^31^ on the contrary, *O. telenomicida* females seem to only exploit volatile kairomones.^32^ However, *O. telenomicida* dominates multiparasitism via a physiological suppressive mechanism mediated by the venom injected inside the host egg by the ovipositing female.^33^, ^34^ Nonetheless, collection of naturally laid egg masses revealed that, in the field, *T. basalis* achieved a higher impact on *Nezara viridula* eggs than *O. telenomicida* although the former parasitoid species suffers from intrinsic competition.^29^ Thus, competition assays under laboratory conditions need to be coupled with field investigations to confirm that the outcome of competitive interactions observed in non‐natural conditions can be validated in more relevant ecological environments.

So far, studies on competitive interactions between *T. basalis* and *O. telenomicida* have not taken into account the possible differences in the parasitoid reproductive traits when exploiting the shared host *N. viridula*. Indeed, reproductive traits, such as number and size of ovarian eggs as well as host handling time, are important factors that can determine the outcome of extrinsic competition^5^ and allow the coexistence of inferior intrinsic competitor.^25^, ^35^ This aspect is important to consider as the shared host, the pest *N. viridula*, is one of the species within the Pentatomidae family capable of producing larger egg masses which are not fixed in terms of clutch size.^36^, ^37^ In fact, in Sicily, the naturally laid egg masses of *N. viridula* are often composed by 40–90 individual eggs (AC, personal observations). Yet competitive interactions investigated so far have focused on intrinsic competition^26^, ^33^, ^34^ and to reach this goal, they have been carried out using a limited number of host eggs which do not reflect the clutch size of naturally laid egg masses and do not allow to investigate the full reproductive potential of the parasitoid species. Nonetheless, Peri *et al*.^29^ have already suggested that interspecific intrinsic interactions between *T. basalis* and *O. telenomicida* in the field are likely to be less severe than in the lab due to the clutch size of naturally laid *N. viridula* egg masses.

In this paper, we carried out experiments to determine: (i) the role played by *N. viridula* host egg mass size in mediating the outcome of extrinsic competition between *T. basalis* and *O. telenomicida* under laboratory conditions, (ii) the reproductive potential of the competing parasitoids species by counting the number and the size of ovarian eggs and recording the host handling time. Finally, manipulative field experiments to investigate (iii) the role played by egg mass size in shaping extrinsic competition between the two parasitoid species have been carried out.

## 2 MATERIALS AND METHODS

### 2.1 Insect rearing

*Nezara viridula* colony was held in wooden cages (50 × 30 × 35 cm), ventilated with mesh‐covered holes (5 cm in diameter), in an environmental room (24 ± 1 °C, 70 ± 5% RH, 16 h:8 h L:D). Bugs were fed with a diet of seasonal fresh vegetables and sunflower seeds. Food was changed every 2–3 days, and separate cages were used for nymphs and adults. Paper towels were placed inside each adult cage as an ovipositional substrate. Daily collected egg masses were used to maintain the stink bug colony which was also regularly refreshed with field‐collected bugs.

The *T. basalis* and *O. telenomicida* colonies were established from wasps emerging from naturally laid *N. viridula* egg masses collected from cultivated tomato fields and surrounding un‐cultivated areas. Adult parasitoids were reared in 16 ml glass tubes (density = 50–60 wasps/tube), fed with a solution of honey–water, and kept in an incubator at the same environmental conditions described for the stink bug colony. Collected *N. viridula* egg masses from the colony were exposed to parasitoids for 48 h, then the wasps were removed, and the parasitized eggs stored for incubation.

### 2.2 Laboratory bioassay

The objective of the experiment was to investigate if the host egg mass size plays a role in egg parasitoid emergences when competing species were released either singly or simultaneously. In all the laboratory bioassays, females of *T. basalis* and *O. telenomicida* were 4–5 day old, mated, and had no previous experience in terms of host oviposition. Wasps were used only once. About 24 h before the experiments, wasp females were isolated in small vials (1.5 × 5 cm) with a drop of honey–water solution. Then, about 1 h before bioassays, they were transferred into the experimental room (24 ± 1 °C, 60 ± 10% RH). The experimental arenas consisted of a small Petri dish (4 cm in diameter) placed inside a larger Petri dish (6 cm in diameter). The inner Petri dish contained, at the center, an egg mass of *N. viridula*, which was up to 24 h old. The egg mass was directly put on the bottom of the Petri dish. The egg mass size was experimentally manipulated to consist of 10, 20, 30, or 40 individual eggs arranged approximately in hexagonal patterns. Four small and equidistant holes (~3 mm diameter) were drilled in the lid of the inner Petri dish to allow the wasps to leave the arena after patch exploitation.

Experiments were conducted in the arena described above according to the following releases: (i) a single *T. basalis* female was introduced in the inner Petri dish (Tb), (ii) a single *O. telenomicida* female was introduced (Ot), and (iii) one female of each species was simultaneously introduced (Tb + Ot). All combinations were replicated 10 times for each of the four different egg mass sizes. The experiment started when a female displayed an ovipositing posture after contacting the egg mass.^26^ When this situation did not occur during 10 min from release into the arena, the trial was discarded. Arenas were checked at regular time intervals (every hour within the first 8 h period, and at 8 h intervals thereafter) and wasps were removed when they were found on the outer Petri dish. After wasps were removed, the parasitized egg masses were placed into incubators (24 ± 1 °C, 70 ± 5% RH, 16 h:8 h L:D) until the emergence of the parasitoids or the eclosion of *N. viridula* nymphs. In all the experiments, wasps were found on the outer Petri dish within 24 h.

### 2.3 Reproductive potential of parasitoids

#### 2.3.1 Host handling time

A subset of parasitoid females that emerged within 24 h from the treatments ‘Tb’ and ‘Ot’ (egg mass size = 20 eggs), was randomly selected to investigate the host handling time, i.e. the time needed for the wasps to complete an oviposition bout. These wasps were offered an egg mass inside a small Petri dish (4 cm diameter) and the host handling time was scored for the first successful parasitism event. A video camera (Zeiss Axiocam ERC) mounted on a stereoscope (Zeiss Stereo Discovery.V12) was used to record the wasp behavior with the aid of AxioVision SE64 Rel. 4.9.1 software for image acquisition and analysis. The time taken by *T. basalis* and *O. telenomicida* to perform the following behavioral steps during an oviposition bout was recorded: (i) tapping the host egg with the antennae (= drumming), (ii) puncturing the host chorion with the ovipositor (= drilling), (iii) withdrawing the ovipositor while staying motionless on the host egg (= resting), (iv) drinking droplets of ooplasm oozing from the punctured host egg (= host‐feeding), (v) inserting the ovipositor to lay egg inside the host (= oviposition), and (vi) sweeping the ovipositor on the surface of the host egg (= marking). For both parasitoid species 10 replicates were carried out.

#### 2.3.2 Size and number of oocytes

Another subset of randomly selected *T. basalis* and *O. telenomicida* females that emerged within 24 h from the 20 eggs treatments was used to assess the egg load of the wasps. Parasitoids were first killed at −18 °C and then dissected in a glass tray filled with phosphate buffered saline (PBS) to count the total number of mature oocytes and estimate the length of the five largest oocytes per individual female. Observations were carried out under a stereomicroscope (Olympus SZX12 with a WHS15\u00D7/ 16 ocular and a De Plapo 1.2 XPF lens; Olympus, Tokyo, Japan) equipped with a calibrated cross‐hair micrometer. Oocytes were counted with the aid of a mechanical cell counter, and the longitudinal distance of the oocyte body was measured as a proxy of size without taking into account the stalk (Figure S1). The average oocyte length per female was used in statistical analyses to eliminate pseudoreplications.

### 2.4 Field experiment

To investigate the role played by egg mass size in shaping extrinsic competition between parasitoid species, a manipulative field experiment was carried out. Three‐week‐old tomato plants (cultivar ‘Costoluto genovese’) were transplanted into the field with 1 × 1 m spacing between plants and allowed to adjust to field conditions for 1 week. The experimental plot consisted of 20 tomato plants. Each plant was covered with a fine‐mesh net to avoid herbivore infestations to occur. After a week, the nets were removed and *N. viridula* egg masses were attached onto the plants according to the following treatments: 1) half of the plants received egg masses artificially assembled to consist of 10 eggs (‘Small’ egg mass); 2) half of the plants received egg masses artificially assembled to consist of 40 eggs (‘Large’ egg mass). Egg masses were produced under laboratory conditions and exposed to the field when they were less than 24 h old. Egg masses were first attached to a piece of cardboard (2 × 4 cm) using Metylan Normal wallpaper paste glue (Henkel, Dusseldorf, Germany) and then placed onto the lower side of leaves with the aid of clip cages. One egg mass was attached to each plant. Eggs were recollected after 5 days of exposure to the parasitoid communities in the field. Subsequently, they were individually kept in the laboratory into in 85 ml glass vials that were closed with cotton wool. The glass vials were checked daily for eclosion of stink bug nymphs or emergence of parasitoids. The experiment was repeated three times during June–September 2019.

### 2.5 Statistical analyses

The number of parasitoids emerged in laboratory and field conditions was tested using Generalized Linear Models (GLMs) with Poisson error distribution and log link function. Parasitoid species, egg mass size and their interaction were fitted as factors in the GLMs using the number of parasitoids emerged as response variable. The number of oocytes produced by *T. basalis* and *O. telenomicida* was compared using a GLM with Poisson error distribution and log link function, whereas the differences in oocyte size were analyzed with a Linear Model (LM) assuming normal error distribution. When over dispersion was detected in the Poisson GLMs, standard errors were corrected using a quasi‐GLM model where the variance is given by *φ* × *μ*, where *μ* is the mean and *φ* the dispersion parameter.^38^

Data from the host handling experiment did not meet parametric test assumptions (e.g. heteroscedasticity) and thus the time spent in different behaviors by *T. basalis* and *O. telenomicida* was compared using U‐Mann–Whitney tests.

Significance of the factors in the GLMs was derived with Likelihood‐Ratio Tests^39^ and post‐hoc comparison was performed using the function *ghlt* in the *multcomp* package.^40^ Significance of the factor oocyte size in the LM was derived directly from *F*‐test.^39^ Model fit of LM and GLMs was assessed with residual plots.^39^ All statistical analyses were processed using the statistical software R version 3.6.3.^41^

## 3 RESULTS

### 3.1 Laboratory bioassay

**Figure 1:**
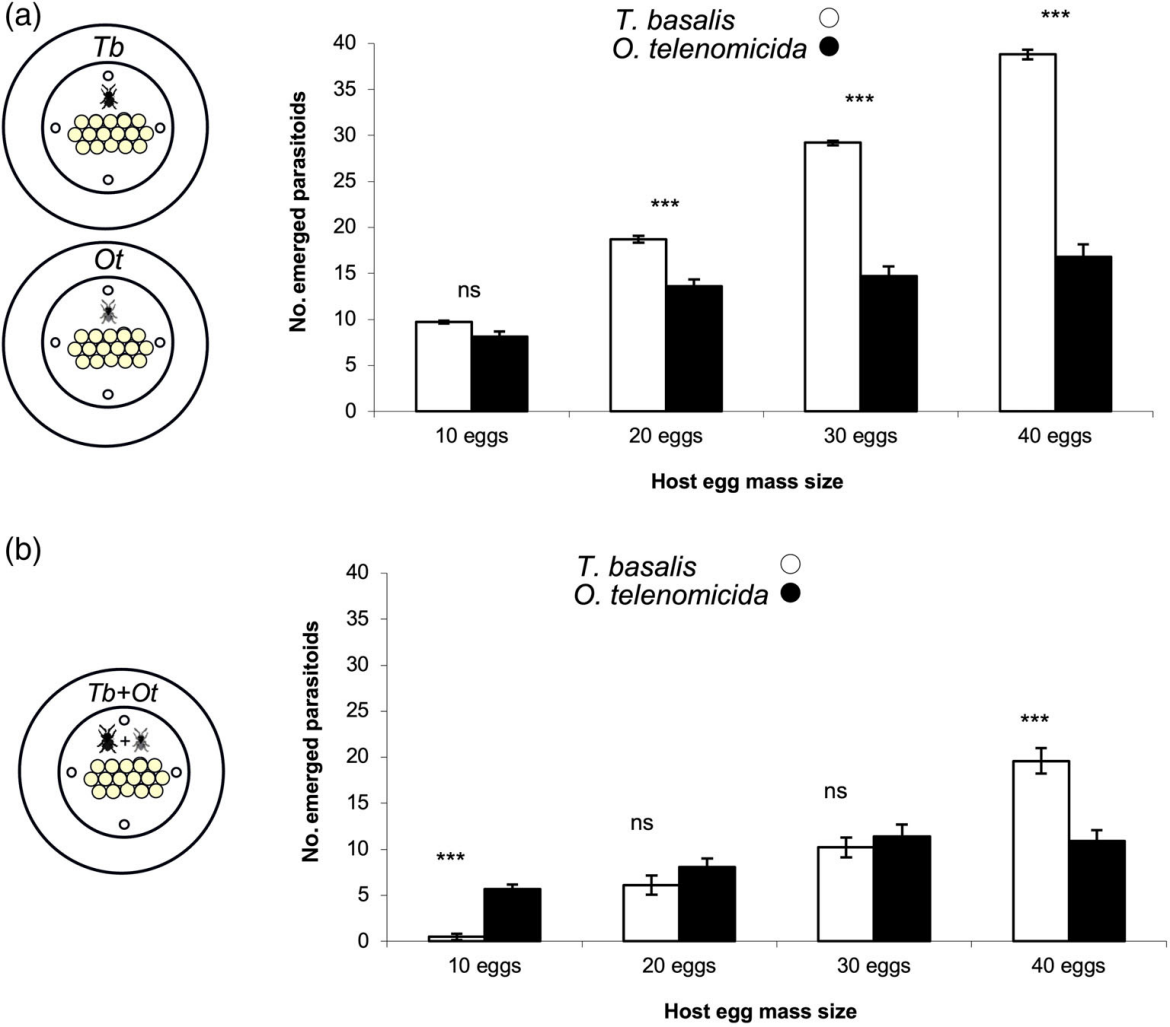
Mean number (± SE) of emerged parasitoids observed from *Nezara viridula* egg masses exposed to parasitoids under laboratory conditions. Egg masses consisted of 10, 20, 30, or 40 individual eggs. (A) single release: the egg mass was exposed either to a *Trissolcus basalis* female or a *Ooencyrtus telenomicida* female; (B) simultaneous release: the egg mass was exposed concurrently to a *T. basalis* female and a *O. telenomicida* female. White bars indicate number of emerged *T. basalis* adults while black bars indicate number of emerged *O. telenomicida* adults. Asterisks above bars indicate significantly different means between *T. basalis* and *O. telenomicida* for the same host size treatment (GLM, *p* < 0.05), ns: not significant.

In the single release, the number of emerged parasitoids was found to be significantly affected by the parasitoid species (GLM, χ^2^ = 341.37; df = 1; *p* < 0.001), by the egg mass size (χ^2^ = 600.47; df = 3; *P* < 0.001) and by the egg mass size × species interaction (χ^2^ = 57.37; df = 3; *p* < 0.001). When more than 20 eggs were exposed, the parasitism was higher in *T. basalis*, while no difference between *T. basalis* and *O. telenomicida* was shown for the 10 eggs treatment (Fig. 1(A)).

When both species were released simultaneously (Tb + Ot), a significant effect of the egg mass size (χ^2^ = 147.23; df = 3; *p* < 0.001) and the egg mass size × species interaction effect (χ^2^ = 61.56; df = 3; *p* < 0.001) was found on parasitoid emergence. No overall effect of the parasitoid species *per se* was found (χ^2^ = 0.010; df = 1; *p* = 0.922). The 10 eggs treatment was favorable to *O. telenomicida* over *T. basalis* in terms of number of emerged parasitoids while the opposite was found for the 40 eggs treatment. A similar response from two parasitoids species was obtained in the intermediate egg mass size treatments (20 and 30 eggs) (Fig. 1(B)).

### 3.2 Reproductive potential of parasitoids

#### 3.2.1 Host handling time

The sequence of oviposition for *T. basalis* consisted of the following steps: (i) drumming, (ii) drilling, (iii) oviposition, and (iv) marking. The parasitoids were never observed to perform host‐feeding, nor resting motionless during an oviposition bout. On average, a *T. basalis* female took 325 ± 9 s to complete an oviposition bout (Table 1). The most time‐consuming step was drilling (196 ± 8 s). The sequence of oviposition for *O. telenomicida* consisted of the following steps: (i) drumming, (ii) drilling, (iii) resting, (iv) concurrent host‐feeding and (v) oviposition. The average time required to successfully lay an egg by a naïve *O. telenomicida* female was 4812 ± 66 s with host‐feeding being the most time‐consuming step to perform (2977 ± 55 s) (Table 1). Females of *T. basalis* spent significant less time compared with *O. telenomicida* in performing the following behavioral steps: drumming (*z* = 3.70; df = 18; *p* < 0.001), drilling (*z* = 3.77; df = 18; *p* < 0.001) and oviposition (*z* = 3.75; df = 18; *p* < 0.001). As a result, the total time taken by *T. basalis* to complete an oviposition bout was by far much shorter than the time taken by *O. telenomicida* (*z* = 3.78; df = 18; *p* < 0.001).

**Table 1:** Time taken and behavioral steps observed by *Trissolcus basalis* and *Ooencyrtus telenomicida* to parasite a *Nezara viridula* egg. Mean (±SE) number of seconds spent drumming, drilling, resting, host‐feeding oviposition and marking. Host handling time was scored for the first successful parasitism event. Different letters indicate significant differences within the same behavioral step (U‐Mann–Whitneytest, *p* < 0.05)

Host handling time	*Trissolcus basalis*	*Ooencyrtus telenomicida*
Drumming	34 ± 3 a	89 ± 11 b
Drilling	196 ± 8 a	1521 ± 189 b
Resting	‑	313 ± 37
Host‐feeding	‑	2977 ± 551
Oviposition	81 ± 8 a	182 ± 17 b
Marking	14 ± 1	‑
Total (seconds)	325 ± 9 sec a	4812 ± 668 sec b

#### 3.2.2 Size and number of oocytes

The average number of *T. basalis* oocytes was significantly higher than the average number of *O. telenomicida* oocytes (GLM, χ^2^ = 316.57; df = 1; *p* < 0.001). The size of the oocytes was also largely different between the parasitoid species, with *O. telenomicida* producing significantly larger oocytes compared with *T. basalis* (LM, *F* = 1485.10; df = 1; *p* < 0.001) (Table 2, Figure S1).

**Table 2:** Number (mean ± SE) and length (mean ± SE) of oocytes produced by *Trissolcus basalis* and *Ooencyrtus telenomicida* females emerged from *Nezara viridula* eggs. Different letters indicate significant differences (GLM, *p* < 0.05)

	*Trissolcus basalis*	*Ooencyrtus telenomicida*
Oocyte number	76.2 ± 2.4 a	24.8 ± 1.6 b
Oocyte length (mm)	109 ± 10 mm a	185 ± 33 mm b

### 3.3 Field experiment

**Figure 2:**
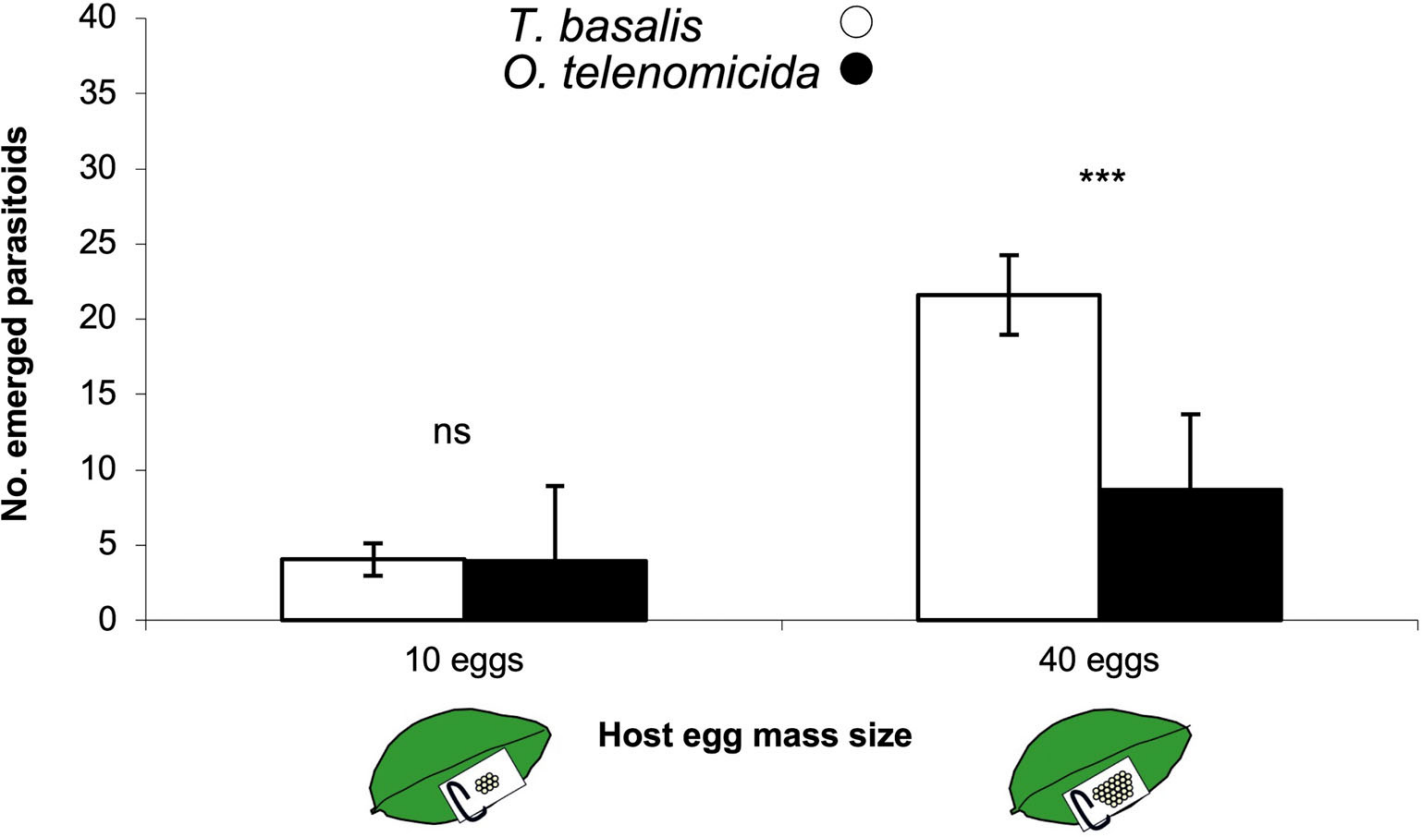
Mean number (± SE) of emerged *Trissolcus basalis* (white bars) or *Ooencyrtus telenomicida* (black bars) observed from *Nezara viridula* egg masses exposed in the field. Egg masses were produced under laboratory conditions and consisted of 10 individual eggs (i.e. ‘Small’ egg mass) or 40 individual eggs (i.e. ‘Large’ egg mass). Asterisks above bars indicate significantly different means between *T. basalis* and *O. telenomicida* for the same host size treatment (GLM, *p* < 0.05), ns: not significant.

In the field experiment, a significant effect of the parasitoid species (GLM, χ^2^ = 14.66; df = 1; *p* < 0.001) egg mass size (χ^2^ = 46.18; df = 1; *p* < 0.001), was found on parasitoid emergence. The egg mass size × species interaction effect was found to be marginally significant (χ^2^ = 3.84; df = 1; *p* = 0.050). No significant differences were found between *O. telenomicida* and *T. basalis* in terms of number of parasitoids emerged from the ‘Small’ egg masses. On the contrary, for the ‘Large’ egg mass size treatment, we found that the number of *T. basalis* emerged was significantly higher than *O. telenomicida* (Fig. 2). No other parasitoid species emerged from the exposed egg masses.

## 4 DISCUSSION

In this work we demonstrated that the size of the host egg mass, expressed as the number of individual eggs that compose the egg mass, has an important role in mediating co‐occurrence of competing egg parasitoid species. It is remarkably to note that *T. basalis*, which is inferior to *O. telenomicida* in terms of larval competition,^26^ is actually the species capable of inflicting the highest mortality to large *N. viridula* egg masses, thanks to its superior abilities in terms of the reproductive traits studied. From a biological control perspective, it is thus important to evaluate potential mismatches between intrinsic and extrinsic competitive abilities as the latter can have an overriding effect for pest control.^12^

Under laboratory conditions we show that *O. telenomicida* almost excluded *T. basalis* when egg masses made of 10 eggs were simultaneously offered to parasitoid wasps. This outcome is likely due to the fact that both species were able to exploit all the available hosts in the treatment composed of 10 eggs only, and due to strong asymmetries in intrinsic competition, *O. telenomicida* almost always emerged from multiparasitized host eggs.^26^, ^33^ In the absence of competition, both parasitoid species exploit equally well such host resources under our experimental conditions. Our results are supported by competitive investigations between *Trissolcus brochymenae* (= *murgantiae*) (Ashmead) and *Ooencyrtus johnsonii* (Howard), two egg parasitoids of *Murgantia histrionica* (Hahn). In fact, the egg mass size of naturally laid egg masses in this stink bug species is generally fixed at 12 eggs and, due to this short host supply, the superior intrinsic species, *O. johnsonii*, can exclude the competitor *T. brochymenae* (= *murgantie*) in laboratory experiments.^42^ However, our results show that the strength of parasitoid competition is affected by the size of the host egg mass. In fact, when larger *N. viridula* egg masses were offered to *T. basalis* and *O. telenomicida* it became clear that these egg parasitoid species largely diverge in terms of host exploitation abilities: a *T. basalis* female was able to exploit efficiently the available egg mass, regardless of the number of eggs it was composed of; on the contrary, a *O. telenomicida* female did not exploit all the available eggs in treatments composed of 20–40 eggs masses. Because of such differences in exploitation abilities, the outcome of competition was reversed in the largest egg masses (40 eggs), with more *T. basalis* adults emerging compared with *O. telenomicida*, even if the former suffered from intrinsic competition in multiparasitized *N. viridula* eggs.

The contrasting exploitative abilities of the competing parasitoid species can be explained considering their different attack rate: the average host handling time required by to successfully exploit the first host egg by naïve *T. basalis* females was about 5 min which confirmed findings by Bin *et al*.^43^ On the contrary, the average host handling time in *O. telenomicida* females was much longer, as more than 1 h was needed to successfully parasitize the first *N. viridula* egg. The functional response of a *O. telenomicida* female shows a saturation in terms of parasitism rates already when egg masses composed of 20 eggs were offered to the wasps, thus the proportion of hosts that avoid parasitism could increase with the egg mass size. Interestingly, *O. telenomicida* always performed concurrent host‐feeding,^44^ a behavior ‐ absent in scelionids such as *T. basalis* ‐ which consists of consuming droplets of host ooplasm before laying its offspring inside the host egg. Other *Ooencyrtus* species are also known to perform host feeding and require a considerable amount of time to successfully parasitize a host.^45^, ^46^

In addition to showing differences in host handling time, wasps also diverged in terms of egg load and type of eggs matured: while the egg load of the proovigenic *T. basalis* was always exceeding the maximum number of host eggs offered to the wasp, this was not the case for the synovigenic *O. telenomicida*. Furthermore, while *T. basalis* develops yolk deficient eggs, *O. telenomicida* matures eggs which are more costly to produce as they are larger and rich in reserves in order to support the embryo development.^47^ Taking into account the different reproductive traits of these egg parasitoid species, it is possible to hypothesize the following exploitation strategies: (i) *T. basalis* females maximize their fitness by exploiting all suitable host eggs because of the large egg load and low egg production costs which compensate for the risk of wasting eggs in the event that mutiparasitism occurs, (ii) females of the intrinsically superior *O. telenomicida* exploit only a limited fraction of all the suitable hosts encountered, because of the low egg load and high costs of egg production. In order to maximize fitness, it is thus important for *O. telenomicida* that most eggs laid will successfully develop, even if multiparasitism occurs. Interestingly, *O. telenomicida* may also prefer to leave the egg mass unexploited even if mature eggs are still available as also observed for *Ooencyrtus nezarae* Ishii females attacking *Megacopta punctatissimum* (Montandon) host eggs.^48^ This peculiar strategy to ‘not lay all the eggs in one basket’ is common in other egg parasitoid species such as *Anagrus delicatus* Dozier when oviposition investments are costly and the mortality risks in the environments are unpredictable.^49^

In general, the competitive patterns found in field conditions were consistent with laboratory investigations indicating that the egg mass size was indeed found to be an important predictor of co‐occurrence of parasitoid species in more relevant ecological settings. Nevertheless, no competitive exclusion was found in field conditions in egg masses composed of 10 eggs (a treatment that favors strong intrinsic competition), probably because *T. basalis* is able to ‘escape’ competition by locating more egg masses compared with the superior larval competitor *O. telenomicida*. Although we were able to score the fate of each host egg, we could not track the oviposition events that occurred when egg masses were exposed in the field. Further investigations with the aid of molecular tools can be instrumental to confirm our hypothesis and unravel how common multiparasitism is in nature.^50^, ^51^, ^52^ The efficient host location strategy adopted by *T. basalis* to locate *N. viridula* egg masses is based on the exploitation of several chemical cues, some of which, such as oviposition‐induced plant volatiles, are reliable predictors of host egg availability on the plant.^31^ Because *T. basalis* females possess superior extrinsic abilities both in terms of host finding and host exploitation, it is likely that *O. telenomicida* females discover egg masses already parasitized by the interspecific competitor in nature. In fact, to co‐exist with *T. basalis*, *O. telenomicida* has established intraguild interactions which are not limited to competition as the latter can even become a facultative hyperparasitoid of the former species.^53^ However, it is important to point out that, in the field, the location of host egg masses depends not only by individual searching abilities, but also by the population density of the parasitoids.

Parasitoid biologists have often discussed that the superior dispersal and reproductive abilities of weak larval competitors are important aspects to consider in species coexistence. For example, already in the 70′, Zwolfer^7^, ^54^ argued that coexistence of parasitoid species attacking forest pests may be facilitated by differences in the relative competitive abilities. He suggested that coexistence may be possible if inferior larval competitors evolved superior abilities at the adult stage in host finding, dispersal or reproduction (the counterbalanced competition principle) and classified parasitoids of forest pests in ‘intrinsically’ or ‘extrinsically’ superior competitors. The results of our study suggest that *T. basalis* and *O. telenomicida* have biological requirements that could fit the counterbalanced competition principle.

In conclusion, the presence of a superior intrinsic competitor such as *O. telenomicida* does not appear to interfere strongly with the biological control efficiency of *T. basalis*, as both parasitoid species display distinctive ways to exploit *N. viridula* egg masses thus reducing the degree of niche overlap. This degree of overlap is further decreased by the fact that the two parasitoid species also exhibit different patterns of temporal occurrence as field surveys have revealed that *T. basalis* is generally present earlier (and for longer) than *O. telenomicida* during the crop growing season.^29^ We argue that studying intrinsic competition in the laboratory is a useful and convenient first step to obtain preliminary information between competing parasitoid species but further evaluations under relevant ecological conditions are needed to truly understand the role of competition in basic and applied ecology.^5^ In particular, our study points out that extrinsic competitive abilities are very important to consider in biological pest control, especially when multiple candidate species are under consideration or when there are possibilities that the candidate biological control agent will interact with resident parasitoid species.

## Supporting information


**Figure S1.** Representative light microscopy pictures of oocytes found in competing egg parasitoid species of the stink bug *Nezara viridula*. (A) Oocyte dissected from *Trissolcus basalis* female. (B) Oocyte dissected from *Ooencyrtus telenomicida* female. The size of the oocytes is largely different between the parasitoid species, with *O. telenomicida* producing larger oocytes compared with *T. basalis*. Red arrows indicate the measured length of oocytes.

## Data Availability

The data that support the findings of this study are available from the corresponding author upon reasonable request.
