# Supplementary material for: Contrasting reproductive traits of competing parasitoids facilitate coexistence on a shared host pest in a biological control perspective
Source: Pest Manag Sci. 2022 May 26;78(8):3376–83. doi: 10.1002/ps.6965 (PMC9541709; doi:10.1002/ps.6965)
Supplement: Supplementary file 1 — Figure S1. Representative light microscopy pictures of oocytes found in competing egg parasitoid species of the stink bug Nezara viridula. (A) Oocyte dissected from Trissolcus basalis female. (B) Oocyte dissected from Ooencyrtus telenomicida female. The size of the oocytes is largely different between the parasitoid species, with O. telenomicida producing larger oocytes compared with T. basalis. Red arrows indicate the measured length of oocytes. [file PS-78-3376-s001.docx]

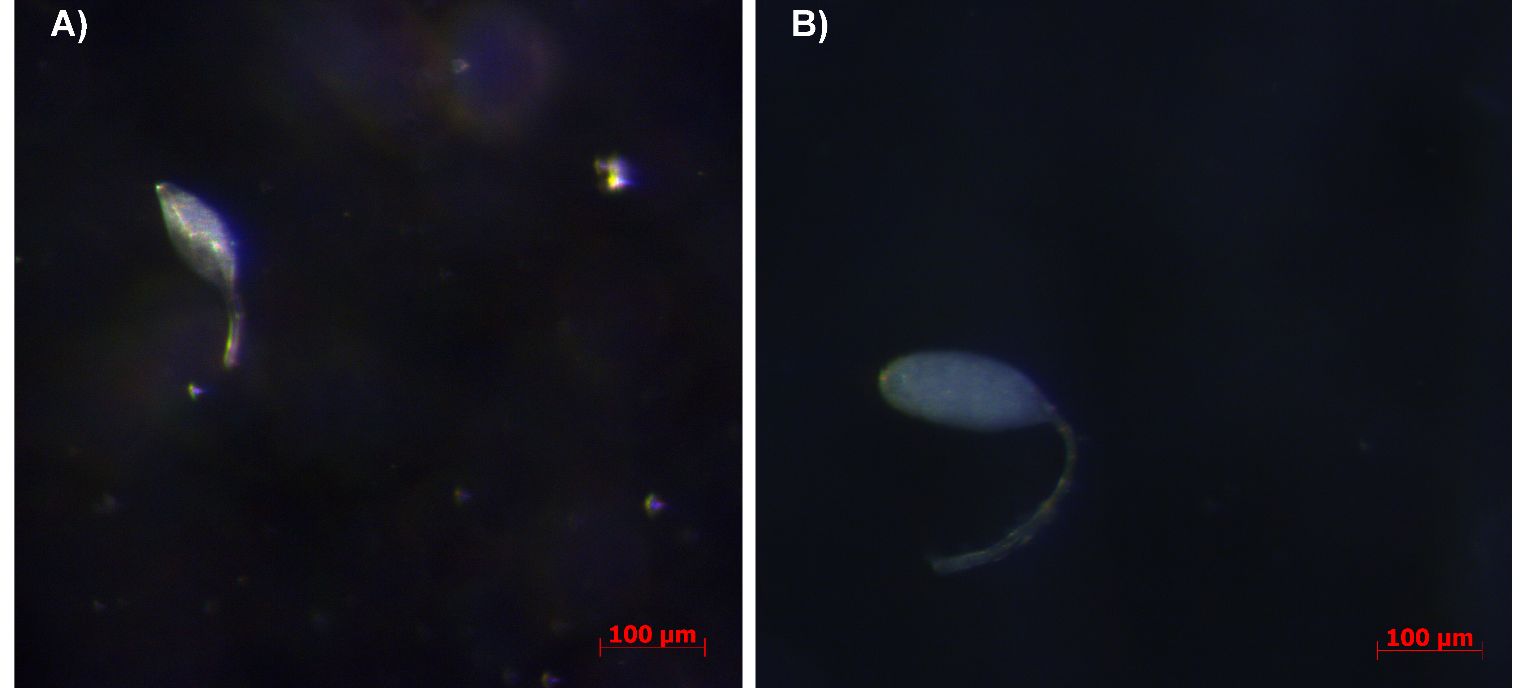


**Figure S1.** Representative light microscopy pictures of oocytes found in competing egg parasitoid species of the stink bug *Nezara viridula*. A) Oocyte dissected from *Trissolcus basalis* female. B) Oocyte dissected from *Ooencyrtus telenomicida* female. The size of the oocytes is largely different between the parasitoid species, with *O. telenomicida* producing larger oocytes compared with *T. basalis*. Red arrows indicate the measured length of oocytes.
